# 2D DIGE Does Not Reveal all: A Scotopic Report Suggests Differential Expression of a Single “Calponin Family Member” Protein for Tetany of Sphincters!

**DOI:** 10.3389/fmed.2015.00042

**Published:** 2015-06-18

**Authors:** Arun Chaudhury

**Affiliations:** ^1^GIM Foundation, Little Rock, AR, USA

**Keywords:** gastrointestinal motility, inhibitory neurotransmission, smooth muscle, phasic, tonic

## Abstract

Using 2D differential gel electrophoresis (DIGE) and mass spectrometry (MS), a recent report by Rattan and Ali (2015) compared proteome expression between tonically contracted sphincteric smooth muscles of the internal anal sphincter (IAS), in comparison to the adjacent rectum [rectal smooth muscles (RSM)] that contracts in a phasic fashion. The study showed the differential expression of a single 23 kDa protein SM22, which was 1.87 fold, overexpressed in RSM in comparison to IAS. Earlier studies have shown differences in expression of different proteins like Rho-associated protein kinase II, myosin light chain kinase, myosin phosphatase, and protein kinase C between IAS and RSM. The currently employed methods, despite its high-throughput potential, failed to identify these well-characterized differences between phasic and tonic muscles. This calls into question the fidelity and validatory potential of the otherwise powerful technology of 2D DIGE/MS. These discrepancies, when redressed in future studies, will evolve this recent report as an important baseline study of “sphincter proteome.” Proteomics techniques are currently underutilized in examining pathophysiology of hypertensive/hypotensive disorders involving gastrointestinal sphincters, including achalasia, gastroesophageal reflux disease (GERD), spastic pylorus, seen during diabetes or chronic chemotherapy, intestinal pseudo-obstruction, and recto-anal incontinence. Global proteome mapping may provide instant snapshot of the complete repertoire of differential proteins, thus expediting to identify the molecular pathology of gastrointestinal motility disorders currently labeled “idiopathic” and facilitating practice of precision medicine.

A recent report (1) addressed a significant but relatively unsolved area of interest to smooth muscle physiologists. Using the advanced proteomics techniques of 2D differential gel electrophoresis (DIGE) and mass spectrometry (MS), the study (1) examined whether differential proteome expression underlies the physiology of tonically contracted sphincteric smooth muscles of the internal anal sphincter (IAS), in comparison to non-sphincteric muscles of the adjacent rectum (RSM, rectal smooth muscles) that periodically contracts in a phasic fashion. The proteomic study revealed that the 23 kDa protein SM22 is present 1.87 fold more in phasic smooth muscles in comparison to tonic smooth muscles (1).

SM22, also called transgelin because of its ability to gel *in vitro* actin, crosslinks actin microfilaments (2). Smooth muscles are unique in that they are able to adjust their contraction/relaxation status by reorganizing the microfilaments of the actin cytoskeleton and the intermediate filament network (3). This whole hypothesis revolves around the central rationale of an ensemble of protein crosslinking in the cell periphery, involving myosin, actin, and the smooth muscle cell membrane, and also potentially with the cytoskeleton. Calponin family members of proteins, including calponin and caldesmon, have been reported to inhibit actomyosin formation by blocking myosin-binding sites on actin, thus reducing cellular mechanical tone (4, 5). SM22 also belongs to the protein family containing “calponin homology domains” (6).

The application of high throughput technologies to addressing these fundamental aspects of gut sphincteric proteomic composition is a unique approach (1). Earlier, global protein profiles of rat jejuno-ileocolic neuro-smooth muscle lysates have identified transgelin (SM22) (7). However, the somewhat incomplete nature of the study report (1) defeats the purpose of the potential impact of implementing a high throughput methodology to delve into a long unanswered question. Surprisingly, previous studies examining the proteome of other tonic muscles of the gastrointestinal tract, like the lower esophageal sphincter (LES), revealed differential distribution of several proteins between the tonic LES and the phasic esophageal body (EB) (8). For example, the EB circular muscle demonstrated higher proportions of (i) LC17a relative to LC17b, (ii) myosin heavy chain isoform with seven-amino acid insert relative to the non-inserted form, (iii) γ-actin relative to α-actin, and (iv)caldesmon (8). This study (8), however, did not find a differential expression of SM22 between phasic and tonic smooth muscles of the esophagus. This observation may be explained by the possibility that there may be diverse musculo-proteome in different gastrointestinal sphincters. However, it is known that SM22 and its differential transcript expression is not well-resolved in a one dimensional gel, but rather shows up in a 2D gel (9), and also has been used in the recent study (1).

Strangely, however, the report (1) lacks in completeness of cataloging the complete repertoire of proteins that are differentially expressed between the phasic and tonic smooth muscles. Only proteins spanning 10–120 kDa are shown, and three spots corresponding to SM22 expression are revealed as the only differential proteins between phasic and tonic smooth muscles (1). No protein spots >120 kDa are demonstrated (1). The identity of the numerous other Cy3/Cy5 and co-localized spots are not revealed (1), leaving the reader to wonder regarding the identity of the differential protein spots. It is unlikely that SM22 is the only differentiated protein between sphincteric and non-sphincteric regions. Earlier studies in the IAS have revealed differential expression of numerous other proteins, namely a composite of kinases/phosphatases, including rho-associated protein kinase II (ROCKII), myosin light chain kinase (MLCK), myosin phosphatase (MYP), and protein kinase C (PKC) (10). These kinases and phosphatases effect a contractile state of myosin with actin and microfilaments, resulting in enhanced cellular mechanical tone (11–13). Remarkably, this recent proteomic study (1) did not identify any of these previously examined enzymatic key components of tone maintenance! The readers await to visualize the datasets when the metadata of the publicly funded study (1) are deposited in PASSEL, PRIDE, or World2D-PAGE.

Polymeric lattice of actomyosin is present in smooth muscles, much like in skeletal muscles, and yet contributes to differential basal tone in the sphincteric regions. This results from precision in regulation of its association. The sphincters function as a sluicegate for luminal flow impedance. The study (1) utilized relatively pure cellular concentrates of IAS and RSM in culture. This is the strength of the study. The presence of SM22 in gastrointestinal smooth muscles has been demonstrated earlier (7, 14). Furthermore, ubiquitous distribution of SM22 in various kinds of mammalian smooth muscles (vascular, uterine, urinary bladder) has been shown (14, 15). These brings into questions of how SM22 exclusively may potentially contribute to development of smooth muscle tone in sphincteric muscles. Three levels of evidence have been provided regarding the differential expression of SM22: (a) 2D DIGE revealed 2.14, 1.64, and 1.82 fold changes of SM22 expression between IAS and RSM, (b) confocal volume imaging of SM22 between IAS and RSM, and (c) quantitative Western blotting of higher expression in the RSM in comparison to IAS (1). The equivocal light microscopic confocal imaging showed diffuse expression of SM22 in both IAS and RSM, and due to differences in volume of the smooth muscles derived from two different regions, it was not possible to decipher the staining expression per unit voxel (1). The advanced proteomic examination, however, revealed the subtle low level of expression of SM22 in the IAS in comparison to RSM (1).

Normally, there are three splice variants of SM22, viz., alpha (α), beta (β), and gamma (γ) (14). In chicken gizzard (phasic smooth muscle), these transcripts have been reported in the ratio of 14:5:1, whereas in vascular smooth muscles, these transcripts are more densely present (including an additional transcript delta, δ) (16). Pig stomach contains all four transcripts (α, β, γ, δ) (14), whereas rabbit urinary bladder contains all the transcripts but in different ratios (15). Likely, the three differential spots identified for SM22 (1) represent these three variants, and thus the fold changes of 2.14, 1.64, and 1.82 represent α, β, and γ, respectively (α being most basic and γ most acidic forms). The staining of SM22 has been done with polyclonal antibody (1), which obviously could not distinguish the distribution of the specific variants.

There may be a few possible reasons for the intriguing observation of subtle differences of SM22 between phasic and tonic smooth muscles. First, these subtle changes could result from differential genomic expression. This is feasible, as genomic programing delineates intermittently spaced sphincteric regions during genesis of the gastrointestinal tracts (17). This may lead to differential gene expression between phasic and tonic smooth muscles. The gene contents and the heterogeneous nuclear RNAs (hnRNA_SM22_) may be examined by Southern and Northern blots. A contrary argument to this possibility is that the regulation may not occur at the gene dosage level. This possibility is also likely because the phasic smooth muscles need to toggle back occasionally to a tonic state of contraction, for example, during propulsion of luminal contents. The inherent state of lower tone than the sphincteric regions is not a rigid phenomenon but subject to physiological variations. This situation gives rise to the second possibility that SM22 differential expression may be regulated at the cytosolic level, for example, by post-transcriptional editing of the mRNA transcripts. It is being increasingly recognized that microRNA plays a major role in different types of muscle formation. For example, DiGeorge’s syndrome related microRNA is needed for vascular smooth muscle phenotypic differentiation (18). Similarly, microRNA modulates formation of cardiac myosin and suppresses smooth muscle type myosin during heart biogenesis (19). MicroRNA-1 has been reported to control SM22 function (20). Whether this is the case may be derived from studies incorporating qRT-PCR and microRNA analysis. Furthermore, SM22 knockout mice have normal gut motility (16). In the vascular smooth muscles, SM22 knockout results in a genetic reprograming to a chondrocytic differentiation pattern resulting in arterial wall calcinosis (21). This may result in increased vascular tone and hypertension. Examining the GI phenotype systematically in SM22 knockout mice will provide valuable insights into its function.

Nearly a century ago, the Fenn effect of dependence on nucleotide availability to change muscle tone according to physiological need was described (22). The role of post-translational modification of SM22 by phosphorylation was tested, and was hypothesized to preclude its protein interaction with actin in the sphincteric smooth muscles (1). The contribution of rho-dependent kinase (ROCK) in this phosphorylation reaction was tested by using a specific pharmacologic inhibitor. Despite earlier reports of increased expression of RhoA-dependent kinase in IAS, sphincteric muscles (10), intriguingly, the 2D DIGE, however, could not identify this difference (1). Gimona et al. have earlier demonstrated that SM22 remains unphosphorylated in unstimulated phasic smooth muscle like the chicken gizzard, as well as after mechanical loading and field stimulation that produces phasic contraction (9). Furthermore, *in vivo* SM22 remain unphosphorylated in tonic muscle like guinea-pig tenia coli (9). ^Dephospho^SM22 was present in contrast to phosphorylated myosin light chain (^phospho^MLC) (9), a major biochemical change that mediates force-generating actomyosin interaction (13). *In silico* analyses using KinasePhos^1^ shows no consensus sequence on SM22 that is sensitive to ROCK, but rather to PKC, which has also been earlier confirmed by *in vitro* analyses (6). The temporal role of PKC and ROCK activation in LES has been previously reported (12). The stoichiometry of particulate PKC has been demonstrated to be lower in the IAS in comparison to ROCK (10). The enzymatic activity of PKC in IAS has been demonstrated in the rabbit IAS (23). Further, though relatively different, both ROCK as well as PKC inhibitors reduce tone in human IAS (24). The present study (1) used pharmacological inhibition of ROCK and PKC and did not find any change of SM22 phosphorylation with the PKC inhibitor. Additionally, the study (1) did not examine^phospho^ SM22-actin binding in the co-immunoprecipitation assays. These discrepancies merits to be addressed in future studies. Innovative tools of actin-SM22 assays may be incorporated, since the overlapping molecular masses of SM22 and IgG light chains (~23 and 25 kDa, respectively) produce confound while detecting SM22 after pull-down with anti-actin antibody. For example, actin antibodies developed in alpaca that lack the immunoglobulin light chain shall help avoid these technical limitations.

Importantly, tonic (as well as phasic) muscles *in vivo* are exquisitely sensitive to cytosolic activation of cyclic GMP (cGMP)/Protein Kinase G, resulting from inhibitory nerve-smooth muscle nitrergic neurotransmission, and remains as the final biochemical steps for stimulus-coupled mechanical relaxation^2^ (25–29). Interaction of cytoskeletal force moderating proteins, like SM22, with the postjunctional signal transduction machinery during basal and stimulated neurotransmission are important areas that may be examined using imaging tools of protein interactions like proximity ligation assay.

The SM22 promoters contain CARG boxes and GC boxes, which are responsive to serum response factor and TGFβ (30–32). Importantly, SM22 is also regulated by the transcription factor “snail” (33). Recently, it has been hypothesized that snail deregulation may contribute to the genesis of pathology involving neuromuscular apparati in diabetes mellitus (34). Whether snail-mediated SM22 functional changes may be contributory to gastrointestinal smooth muscle dysfunction in gastrointestinal motility disorders may be a significant area of future investigation.

Recent evidence suggests that neurotransmitter signals may possess a storage form in the postjunctional cell. The significance of these observations is far from known. For example, S-nitrosylproteome assays show S-nitrosyl-SM22 in myometrium of pregnant guinea-pigs. This may facilitate nitric oxide (NO)-mediated relaxation of myometrial smooth muscles independent of global cGMP elevation or activation of its cognate kinase, protein kinase G type I (35). This may have tremendous impact on the modulation of physiology of gastric emptying in pregnancy. Whether the present observations (1) are extensible to LES, pylorus and ileocolic sphincter, and juxta-sphincteric regions, shall validate the role of SM22 in esophagogastrointestinal motility and role of SM22 in specific disease pathophysiology affecting transit of luminal contents.

Advanced proteomics shall find increasing place in investigating panoramic protein changes in hypertensive sphincters, for example, in achalasia or gastroparesis, or hypotensive sphincters, like the LES in gastroesophageal reflux disease (GERD) or the pyloric sphincter in biliary reflux, or IAS in fecal incontinence or anorectal malformations (36). The cutting-edge technologies of advanced proteomics shall help form rationale approaches to disorders currently labeled as “functional,” including dyspepsia, irritable bowel syndrome (IBS), unexplained constipation, and intestinal pseudo-obstruction (37). It should be noted that not all gastrointestinal motility disorders manifest changes in protein expression and may involve changes in the protein function or post-translational modification(s). For example, prejunctional contents of neuronal nitric oxide synthase (nNOS), the enzyme synthesizing the major inhibitory neurotransmitter NO, may remain unaltered in select disorders of impaired gastrointestinal smooth muscle relaxation like achalasia or diabetic gastroparesis (38, 39). Similarly, future studies should address whether the *in vitro* serum-deprived culture conditions (1) resulted in molecular plasticity of the IAS smooth muscle cells. Calponin is known to decrease during *in vitro* culture, whereas SM22 remains relatively unaffected (9). SM22 expression is sensitive to transcriptional agents present in the serum (32). In fact, deprivation of serum response factors has been correlated with occurrence of chronic intestinal pseudo-obstruction (40).

Full-thickness gastrointestinal muscular biopsies from sphincteric and non-sphincteric regions are difficult to obtain for pragmatic reasons (39). Innovative approaches are needed for routine proteome examination of peripheral fluids or tissues and luminal contents to correlate with actual neurotransmitter contents in the enteric nerve terminal-smooth muscle junctions (40). All of these aspects merit consideration while obtaining “big data” related to chronic pathophysiology affecting gastrointestinal neuromuscular function. The wave of personalized medicine to redefine the molecular pathology of the so-called “idiopathic” disorders of gut motility will find increased clinical applications of the high throughput proteomic techniques and contribute to a top-down view of what lies beneath the often-unexplainable clinical observations, thus enabling to practice precision medicine.

In summary, this current investigation (1) is timely reopening of the Pandora’s Box, delving into mechanisms of understanding how sphincters are formed. Because phasic muscles have the ability to produce agonist-mediated maximal contraction similar to tonic muscles, and tonic muscles possess ability to relax to baselines comparable with that of phasic muscles during inhibitory neurotransmission, the fine regulation of multiplex proteins likely results in this complex regulation. The importance of the subtle differences in the SM22 splice variants between sphincteric and non-sphincteric regions, along with the repertoire of other potential contributing proteins, merits detailed future study.

## Conflict of Interest Statement

The author declares that the research was conducted in the absence of any commercial or financial relationships that could be construed as a potential conflict of interest.

## Footnotes

^1^http://kinasephos2.mbc.nctu.edu.tw/

^2^http://www.nature.com/nature/report/index.html?comment=59974&doi=10.1038/464466a
